# Magnetic and near-infrared derived heating characteristics of dimercaptosuccinic acid coated uniform Fe@Fe_3_O_4_ core–shell nanoparticles

**DOI:** 10.1186/s40580-020-00229-4

**Published:** 2020-06-08

**Authors:** Changhyuk Koo, Hwichan Hong, Pyung Won Im, Hoonsub Kim, Chaedong Lee, Xuanzhen Jin, Bingyi Yan, Wooseung Lee, Hyung-Jun Im, Sun Ha Paek, Yuanzhe Piao

**Affiliations:** 1 Program in Nano Science and Technology, Graduate School of Convergence Science and Technology, Seoul National University, 145 Gwanggyo-ro, Yeongtong-gu, Suwon-Si, Gyeonggi-do 16229 South Korea; 2 Department of Neurosurgery, Clinical Research Institute, Seoul National University Hospital, Daehak-Ro 101, Seoul, 110-744 South Korea; 3 Cancer Research Institute Ischemia/Hypoxia Disease Institute Seoul National University College of Medicine, Seoul, Republic of Korea; 4 Advanced Institutes of Convergence Technology, 145 Gwanggyo-ro, Yeongtong-gu, Suwon-Si, Gyeonggi-do 16229 South Korea

**Keywords:** Magnetic nanoparticles, Core–shell nanoparticle, Hyperthermia, Photothermal material

## Abstract

Among the number of hyperthermia materials, magnetic nanoparticles have received much attention. In this work, we studied the heating characteristics of uniform Fe@Fe_3_O_4_ core–shell nanoparticle under near-infrared laser irradiation and external AC magnetic field applying. The Fe@Fe_3_O_4_ core–shell nanoparticles were prepared by thermal decomposition of iron pentacarbonyl and followed by controlled oxidation. The prepared uniform particles were further coated with dimercaptosuccinic acid to make them well dispersed in water. Near-infrared derived photothermal study of solutions containing a different concentration of the core–shell nanoparticles was made by using 808 nm laser Source. Additionally, magnetic hyperthermia ability of the Fe@Fe_3_O_4_ nanoparticle at 150 kHz and various oersted (140–180 Oe) condition was systemically characterized. The Fe@Fe_3_O_4_ nanoparticles which exhibited effective photo and magnetic hyperthermia are expected to be used in biomedical application.

## Introduction

Nanoparticles, such as silica, iron oxide, zinc oxide, and gold nanoparticles, have attracted much research interest for various biomedical applications, for instance, bioimaging [1–4], hyperthermia treatment [5–8] and other therapeutic applications [9–12]. Especially, many kinds of photo and magnetic hyperthermia nanomaterials have been investigated these days. Near-infrared laser and external magnetic field which are normally used in this application are known as less harmful to the human body and possess effective cancer cell killing ability.

Regarding hyperthermia treatment, gold nanoparticles are known as the most effective photothermal material due to the surface plasmon phenomenon which enhancing the photothermal effect. However, the mechanical weakness of gold nanoparticles limited their actual applications in the biomedical field. Therefore, many efforts have been made to prepare new photothermal materials with enhanced mechanical strength [13–16].

Unlike gold nanoparticles which possess a mechanical weakness in photothermal process, most of the magnetic nanoparticles do not show obvious morphology changed during magnetic hyperthermia experiments. In recent years, many kinds of research have been made to prepare magnetic nanoparticles with high magnetic saturation value [17, 18] for more effective heat generation [19–21].

Among various nanomaterials that meet the above purpose, iron nanoparticles are characterized by their inherent strong magnetism and material rigidity. However, iron itself is easily oxidized under air or water exposure condition. As a result, many research works have been made to improve the stability of iron nanoparticles. According to improve the stability of iron nanoparticles, the nanoparticles further coated with other material and carbon, silica or other metal oxide are routinely used as candidate materials [22–25]. Iron oxides as coating materials have attracted much attention due to their low toxicity, good magnetic properties, therapeutic effect and easy synthesis methods [26–29].

In this work, we studied the heating characteristics of uniform Fe@Fe_3_O_4_ core–shell nanoparticle under near-infrared laser irradiation and external AC magnetic field applying. The Fe@Fe_3_O_4_ core–shell nanoparticles were prepared by thermal decomposition of iron pentacarbonyl and controlled oxidation of iron edge [30, 31]. According to the TEM Images, the synthesized nanoparticle size was measured with a total size of 14.9 ± 1.2 nm with core size 9.6 ± 1.1 nm. Vibrating-sample magnetometer shows that the magnetic saturation value of the pristine Fe@Fe_3_O_4_ nanoparticles was measured as 83.3 emu/g. The Fe@Fe_3_O_4_ nanoparticles were further coated with dimercaptosuccinic acid (DMSA) to obtain water dispersity. Photothermal ability study of the coated nanoparticle was conducted at various laser power and concentration. To understand the heating ability of the synthesized nanoparticle via quantitative analysis, heat conversion efficiency was calculated by a simple mathematical method and the calculated efficiency was measured at a maximum of 33.21% and a minimum of 20.79% under various experimental conditions. Moreover, the magnetic hyperthermia test was operated at 150 kHz with various oersted (140–180 Oe) condition. The particles show perfect cycle stability during 5 times repeating the photothermal test.

## Experimental procedure

### Chemicals

Iron pentacarbonyl (Fe(CO)_5_), Oleylamine (OAm, purity 70%), 1-octadecene (ODE, purity 90%), DMSA and hexadecylamine (HDA, purity 95%) were purchased from Sigma–Aldrich. Hydrochloric acid (HCl, purity 35-37%) was purchased from Samchun. Trimethylamine *N*-oxide (purity 95%) were obtained from TCI. Dimethyl sulfoxide (DMSO, purity 90%) were obtained from JUNSEI.

### Characterization

Transmission electron microscopy (TEM) images were carried out by a LIBRA 120 Energy-filtering transmission electron microscope at an acceleration voltage of 120 kV. High-resolution TEM (HR-TEM) image was recorded with JEM-2100F at an acceleration voltage of 200 kV. X-ray diffraction pattern was measured by Buker New D8 Advance model using Cu Kα radiation (λ = 0.15418 nm). Analysis of the X-ray photoelectron spectroscopy (XPS) was performed by NEXSA, ThermoFisher Scientific. Hysteresis loop was obtained from vibrating sample magnetometer (VSM) by Lake shore VSM-7410. The hydrodynamic size distribution and zeta potential data of the Fe@Fe_3_O_4_ measured by dynamic light scattering using a Zetasizer Nano ZS. UV spectrum was investigated by PerkinElmer Lambda 35 UV/VIS spectrometer. The photothermal test was studied by PSU-W-FC laser power supply. The AC magnetic induced heating characteristics of nanoparticles were measured by using a specially designed HF induction generator. HF induction generator consists of AC coils, DC power supplies, water chiller, capacitor, optical thermometers, function generators, and a PC system. This device operates 30-150 kHz of frequencies and 80–200 Oe of magnetic field strength.

### Synthetic route for Fe@Fe_3_O_4_ nanoparticles

After making a mixed solution of 1-octadecene (20 mL) and oleylamine (0.3 mL) in a three-neck flask, 280 mg of HDA·HCl (HDA·HCl were prepared by followed previous research work reported by Lacroix [30]) was further added to the above solution. The solution was then heated to 120 °C under nitrogen atmosphere and kept at that temperature for 30 min to degas the solution. After increase the temperature of the mixture solution to 180 °C, 0.7 mL of Fe(CO)_5_ was added and kept at that temperature for 30 min with stirring. Then, the solution was cooled down to 100 °C and 7.5 mg of Trimethylamine N-Oxide was adding. The solution was further heated up to 250 °C with heating ratio of 10 °C/min and kept at that temperature for 30 min.

### Surface modification of the synthesized nanoparticle

The synthesized Fe@Fe_3_O_4_ particles were washed with ethanol for 3 times and redispersed in 5 ml of toluene. The, 50 mg of DMSA and 5 ml of dimethyl sulfoxide (DMSO) were added to the solution and further stirring for 48 h.

### Laser irradiation and photothermal effect study

To measure the heating characteristics of the Fe@Fe_3_O_4_ core–shell nanoparticles under NIR laser irradiation, 1 ml of Fe@Fe_3_O_4_ core/shell nanoparticle dispersion was added in an optical cuvette and irradiated by a NIR laser at 808 nm. The temperature of the solution was measured by a thermometer for every 30 s. The laser power and the concentration of the solution were tuned during the test to study the relationship of these factors.

### Magnetic hyperthermia effect study under applied AC magnetic field

The heating characteristics of the Fe@Fe_3_O_4_ core–shell nanoparticles were measured by the AC magnetic field. In the middle of the coil, 1 mL of the nanoparticle dispersion was detected by a thermo-optical sensor. The temperature of the solution was measured under magnetic field strength at 140, 150, 160, 170, and 180 Oe with fixed frequency at 150 kHz. The total measured time was 1000 s. We used 600 s for heating the solution, and 400 s for cooling the solution.

## Results and discussion

### Synthesis and characterization of Fe@Fe_3_O_4_ particles

**Scheme 1:**
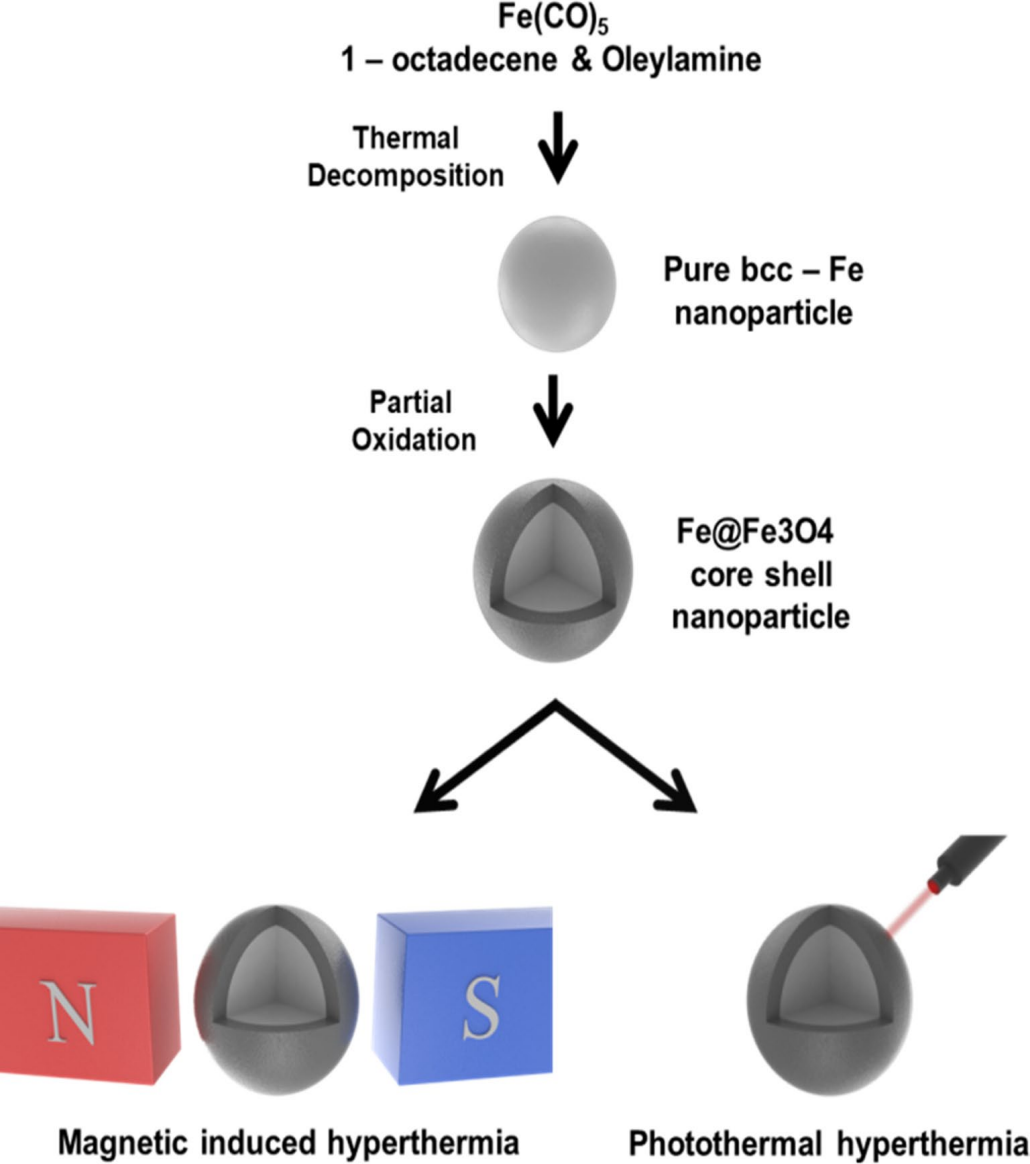
Schematic illustration for the synthesis of Fe@Fe_3_O_4_ core–shell nanoparticle and their hyperthermia application

The Fe@Fe_3_O_4_ core–shell nanoparticles were prepared by thermal decomposition method according to literatures [30, 31]. Schematic procedure for preparing nanoparticle and their hyperthermia application is depicted in Scheme 1. The core–shell nanoparticles were prepared by thermal decomposition of iron pentacarbonyl and controlled oxidation of iron edge. In the presence of 1-octadecene, oleylamine and hexadecyl ammonium chloride at 180 °C under nitrogen atmospheric condition, iron pentacarbonyl was thermally decomposed and composite pure body-centered cubic iron nanoparticle. After the reaction, the synthesized iron nanoparticles were further reacted with trimethylamine *N*-oxide at 250 °C for 30 min for improving stability via making iron oxide shell from the partial oxidation of iron edge.

**Fig. 1:**
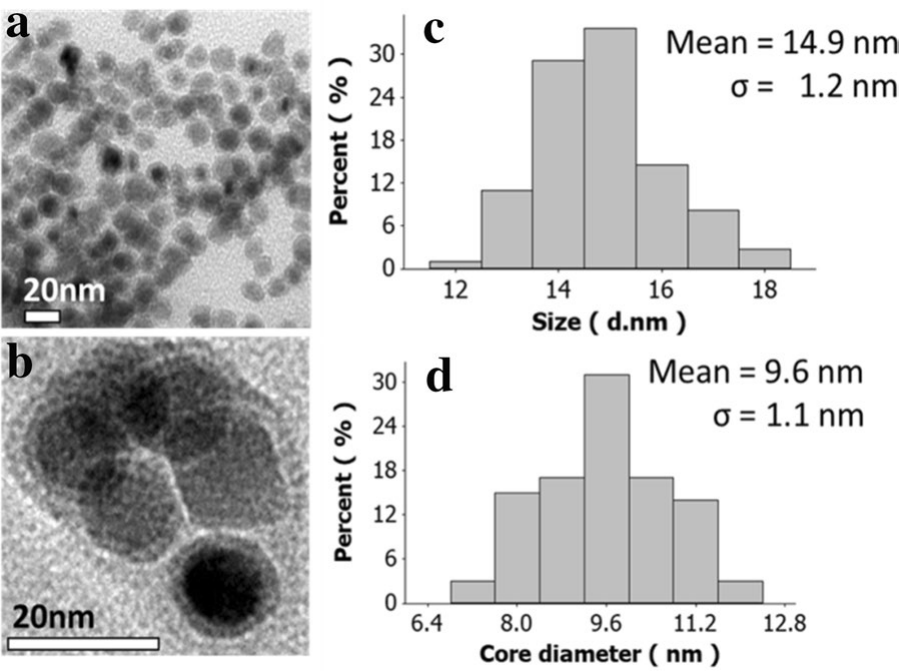
**a** Low and **b** relatively high magnification TEM images of the synthesized Fe@Fe_3_O_4_ nanoparticles prepared via thermal decomposition of iron pentacarbonyl. Size histogram of **c** whole particles and **d** the iron core

Several analytical methods, such as TEM, HR-TEM, VSM, XPS and XRD, was used to characterize the as-prepared nanoparticle. TEM images of the synthesized Fe@Fe_3_O_4_ nanoparticles and their size histogram obtained from the image were summarized in Fig. 1. Because of the different electric conductivity between the iron core and iron oxide shell, certain contrast difference within the nanoparticle could be observed by TEM images.

According to Fig. 1, the average diameter of nanoparticles calculated from TEM image was measured to be 14.9 ± 1.2 nm with a core size of 9.6 ± 1.1 nm. Although the difference between the total and the core size of the synthesized nanoparticles varies great, the standard deviation difference is not significant. This is because the oxidation reaction of core iron was hindered due to the formation of iron oxide shell which prohibited penetration of oxidant into the inner iron core layer. Therefore, after the formation of the certain thickness of the shell, oxidation reaction will not occur anymore and so that the nanoparticles have similar shell thickness. As the results of this phenomenon, the standard deviations of total size and the core size is similar value. We calculate domain size of Fe crystal by Scherrer equation, and the result is 9.9 nm, which is consistent with the core size measured by TEM in Fig. 1d.

**Fig. 2:**
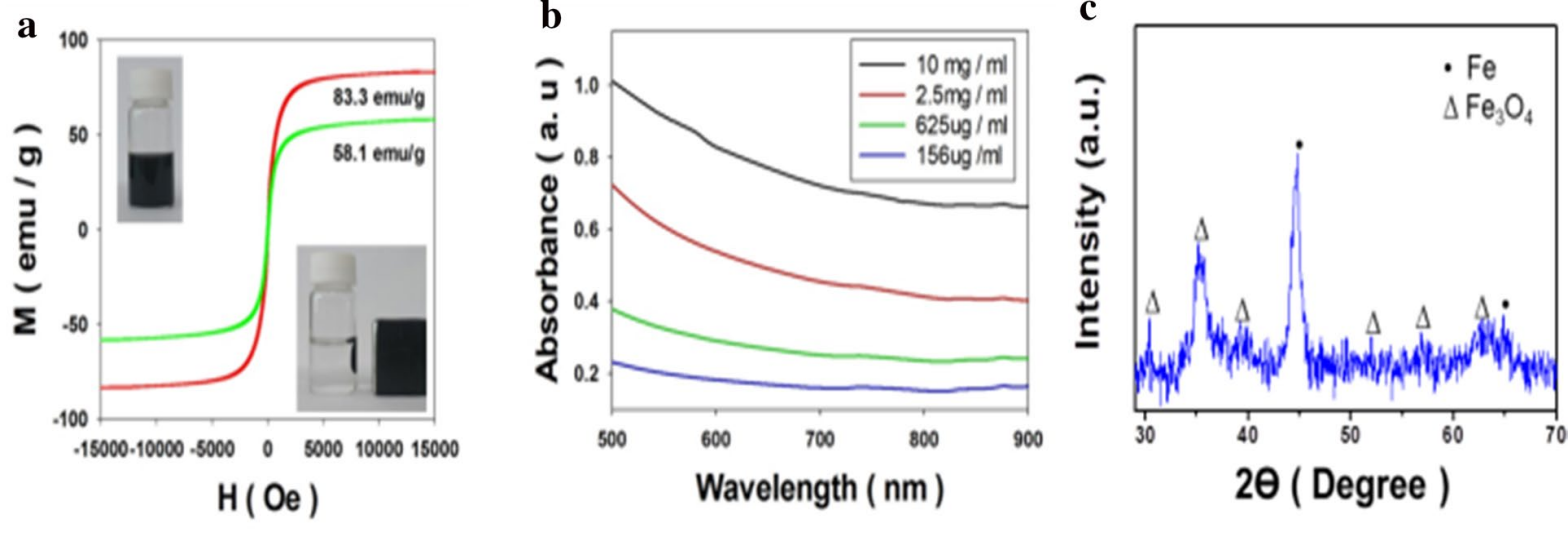
Hysteresis curve of **a** the synthesized Fe@Fe_3_O_4_ nanoparticles (red) and the particles after coated with DMSA (green), insert shows the nanoparticle dispersion response to the outer permanent magnet. **b** Plots of the absorbance spectrum according to the concentration of the DMSA coated particle dispersion, and **c** XRD pattern of the Fe@Fe_3_O_4_ nanoparticle

For understanding of Fe@Fe_3_O_4_ nanoparticles, magnetic properties were characterized using vibrating sample magnetometer (VSM). The results obtained from VSM analysis is shown in Fig. 2a and also the figure contains the images which also shows the response of Fe@Fe_3_O_4_ what dispersed in water by an external permanent magnet. The magnetic saturation of Fe@Fe_3_O_4_ nanoparticles was 83.3 emu/g and this value is similar to that of bulk iron oxide particles. XRD pattern of the Fe@Fe_3_O_4_ nanoparticle is shown in Fig. 2c. The experimental result shows that major peaks of the nanoparticle are well matched with the body-centered cubic Fe (PDF 00-006-0696) and Fe_3_O_4_ nanoparticle (PDF 00-065-0731). This XRD analysis data also supports the fact that the synthesized nanoparticles contain both iron and iron oxide phase.

**Fig. 3:**
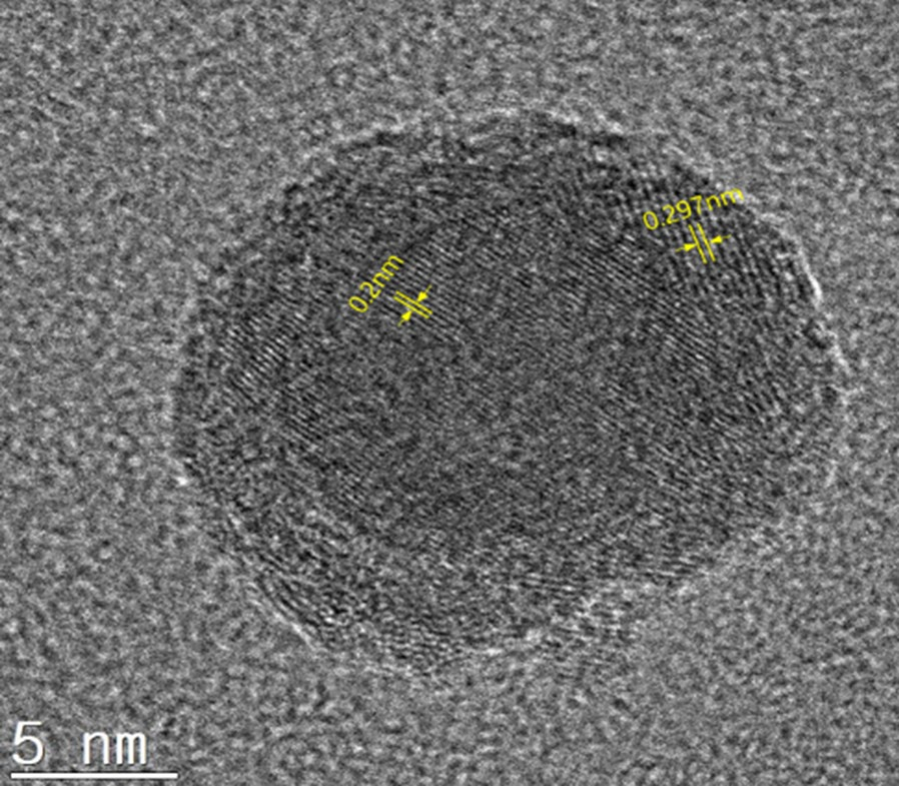
HR-TEM image of the as-prepared Fe@Fe_3_O_4_ nanoparticle

The crystal structure of nanoparticles was also observed by HR-TEM. The 2.0 Å lattice fringe was observed in core which is characteristic of the bcc-Fe (110) lattice planes, and 2.97 Å lattice fringe was observed in shell which is characteristic of the magnetite (220) lattice planes in Fig. 3.

**Fig. 4:**
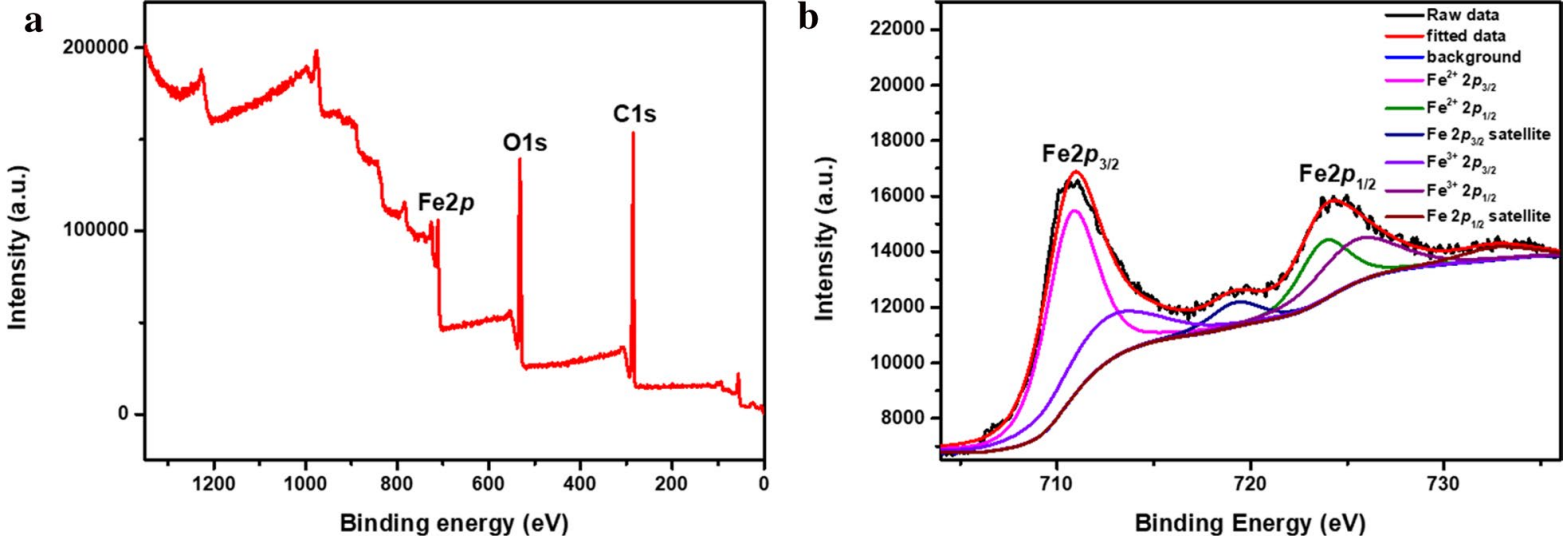
XPS spectra of Fe@Fe_3_O_4_ nanoparticles: survey spectrum (**a**) and Fe 2p XPS spectrum (**b**)

The XPS was carried out to confirm that composition of iron oxide shell. The Fe 2*p* XPS spectrum (Fig. 4b) shows the main peaks of Fe 2*p*_3/2_ at 711.1 eV and Fe 2*p*_1/2_ at 725.0 eV, which accord the typical Fe_3_O_4_ peaks. The XPS peaks observed at the Fe 2*p* core level can be divided into Fe^2+^ peaks (Fe 2*p*_3/2_: 710.7 eV, Fe 2*p*_1/2_: 723.75 eV, and satellite: 719.2 eV) and Fe^3+^ peaks (Fe 2*p*_3/2_: 712.4 eV, Fe 2*p*_1/2_: 725.4 eV, and satellite: 732.67 eV) [32].

### DMSA coating process for water dispersion

DMSA coating process is commonly used to give water dispersivity to the nanoparticles [33–35]. Therefore, the Fe@Fe_3_O_4_ nanoparticles were coated with DMSA to ensure the particle to be well dispersed in water for further experiments. The colloidal stability of the surface coated Fe@Fe_3_O_4_ nanoparticles were investigated by dynamic light scattering (DLS) and zeta potential measurements via Zetasizer Nano ZS. DLS measurements and zeta potential data are shown in Additional file 1: Fig. S1, and the DLS data of the DMSA coated Fe@Fe_3_O_4_ nanoparticles which shows narrow hydrodynamic size distribution indicates that the nanoparticles are well dispersed in distilled water. Moreover, the coated nanoparticles have negative zeta potential value (− 23.5 mV) due to their carboxylate end. Even though the DMSA attached particles have lower saturation magnetization than that of pristine nanoparticle, the saturation magnetization value of coated nanoparticles is steel higher compared to than that of iron oxide nanoparticles which have a similar diameter. After DMSA coating, magnetic property of nanoparticles is decreased in Fig. 2a. The reason is that the weight of the particles increases. Same phenomenon can be seen in other coated magnetic nanoparticles [36, 37].

### Heating performance measurement under Near infrared (NIR) laser irradiation

**Fig. 5:**
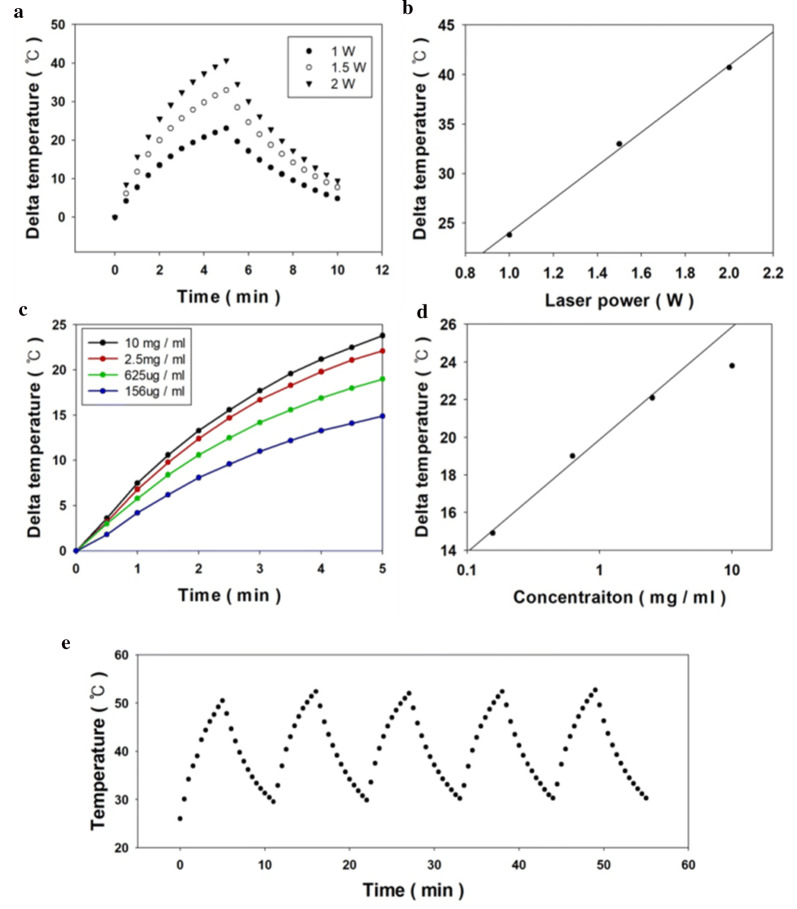
Photothermal characteristics of the synthesized nanoparticles (**a**, **b**) according to the laser power, (**c**, **d**) concentration and (**e**) cycle test (10 mg/ml)

Heating characteristics of Fe@Fe_3_O_4_ under NIR irradiation were investigated using DMSA coated nanoparticle containing a solution. Before the researches about heating characteristic, the light absorptivity of the synthesized nanoparticles was studied for more understanding about the effect of concentration on absorption behavior. The absorption spectrum of the nanoparticle was characterized by Lambda 35 UV VIS spectrometer at 500 to 900 nm wavelength range. The absorption spectrum of the coated nanoparticles which were illustrated in Fig. 2b does not show significant differences from the previous research works about iron oxide nanoparticles [38, 39]. Even though the ordinary absorption characteristics about the infrared region, the synthesized nanoparticle shows effective heating ability during NIR laser irradiation. The temperature of the sample was raised according to the irradiation time, laser power and solution concentration and the details were described in Fig. 5. As a result of Fig. 5a and b, the photo-derived heat generation of the nanoparticles is linearly proportional to the intensity of the applied laser power at the same concentration. This phenomenon shows that the applied laser is absorbed to a certain degree irrespective of the intensity.

Moreover, the response of the nanoparticles to the concentration change is shown in Fig. 5c, d, and the graph as a whole shows the logarithmic form. This phenomenon is thought to be caused by heat exchange of the solution and surrounding and this convection based heat exchange is linearly proportional to the temperature difference between the solution and circumstance air temperature. Therefore, when the temperature change profile is sharply changed at the initial stage of the irradiation and after the certain times when the heat generation and heat loss get similar value the temperature change of the solution is converged.

However, the tendency of the 10 mg/ml condition is not followed these theoretical bases and it exhibited a little different compared to 2.5 mg/ml condition. These phenomena were explained by the intrinsic property of the material which the energy conversion ability from light to heat. Therefore, when the concentration is reached a certain high value, the particles could not transfer more light to heat anymore. Several previous studies have also supported this assumption by showing the phenomenon of temperature saturation at high concentrations [40, 41].

$${\text{Heat generation}} = {\text{C}} \cdot \Delta {\text{T}}$$

$${\text{Efficiency}} = \frac{\text{Heat generagtion}}{\text{Irradated energy}}$$

Total heat generation and heat conversion efficiency were calculated by the simple mathematical method illustrated above and the calculated data were shown in Additional file 1: Fig. S2. Details about the characters in the equation, C is heat capacitance of solution (4.12 J/K) and T indicates the Celsius degree temperature. From the calculated data, the photothermal conversion efficiency was 33.21–20.79% according to the concentration of the solution.

Furthermore, we had conducted experiments to figure out the reusability of the synthesized nanoparticles which shown in Fig. 5e. The experiments were carried out by heating the solution with a concentration of 10 mg/ml for 5 min in 1-watt laser power. The remaining 4 data except for the first cycle showed almost the same tendency and the average conversion efficiency of the simple efficiency calculation was 31.13%. The results of this study are as shown in Additional file 1: Table S1. In order to examine the stability for biomedical applications, Fe@Fe_3_O_4_ nanoparticles were stored in phosphate-buffered saline (PBS) solution for 48 h and then measured for photothermal characteristics (Additional file 1: Fig. S2b). After 48 h, most Fe@Fe_3_O_4_ nanoparticles were settled down. After agitating, the nanoparticles were redispersed (Additional file 1: Fig. S2a). However, the dispersity might be not the same as before. Therefore, the particle density in the laser spot should be lower than the as-prepared sample, which causes slightly lower delta temperature. Given that the performance of the particles is almost maintained, iron oxide shell is successfully protected Fe core from oxidation. Consequently, Fe@Fe_3_O_4_ coated with DMSA maintained stability and magnetic hyperthermia properties after 48 h in PBS.

### Heating performance measurement under external AC magnetic field

**Fig. 6:**
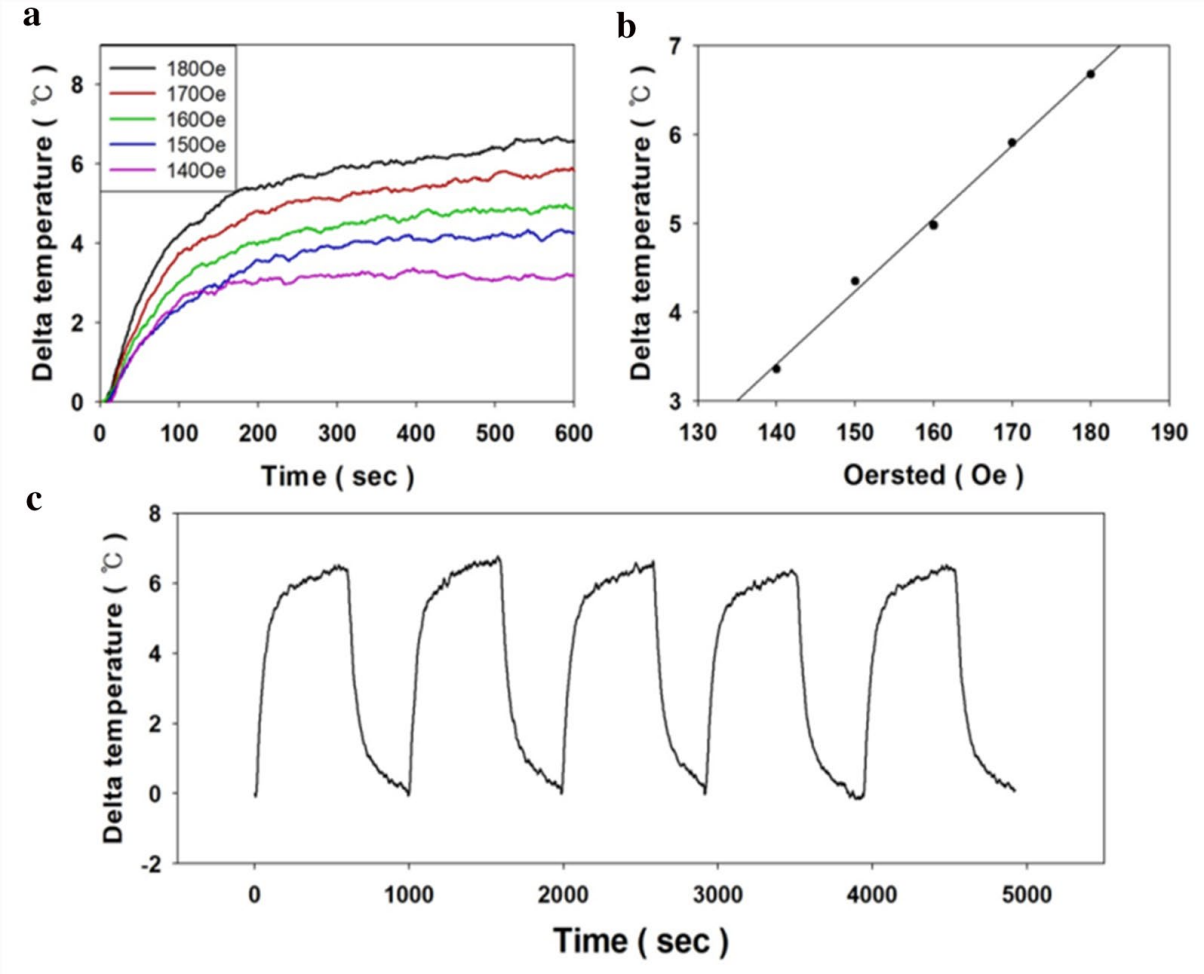
Magnetic hyperthermia characteristics of the synthesized nanoparticles (10 mg/ml) (**a**), according to the strength of external magnetic field (**b**) maximum temperature versus various oersted and their fitting curve (linear reference line) (**c**) cycle test

In order to learn about heating characteristics under the AC magnetic field, we conducted a magnetic hyperthermia experiment at 150 kHz with various oersted (140–180 Oe) and the result is shown in Fig. 6a. The magnetic field with 150 kHz was applied to avoid the local heating by eddy current and its availability for biomedical applications [42]. According to the data, magnetic heat generation was amplified by a stronger external magnetic field. Theoretically, magnetic hyperthermia is caused by two relaxation mechanisms, Neel and Brownian relaxation. Previous studies have suggested that Neel relaxation is a more important factor when magnetic hyperthermia was studied Below 300 kHz [43]. Also, in general, the total heat generated is linearly proportional to the oersted of the external magnetic field [44]. This is due to the fact that the magnetic dipoles of the magnetic nanomaterial are strongly and rapidly aligned by the external electro-magnetic field, and when the AC magnetic field is reversed, there is a correspondingly high degree of alignment to the opposite side. In this process, the spin of the magnetic nanoparticles turns stronger and faster, and as a result, as the intensity of the magnetic field increases, the magnetic hyperthermia effect is promoted.

According to our data illustrated in Fig. 6b shows that the heat generation from the electromagnetic field is directly related to the strength of an external electromagnetic field and these results are well matched with the theoretical bases. Interestingly, delta temperature is much lower than that of NIR-mediated heating. This might be attributed to low magnetic field frequency (150 kHz) and low saturation magnetization of Fe@Fe_3_O_4_ (58.1 emu/g) caused by DMSA coating layer.

Cycle stability under AC magnetic field was also studied and the results are shown in Fig. 6c. The results suggest that Fe@Fe_3_O_4_ core–shell nanoparticles have sufficient strength to withstand the stress from external magnetic fields. In practice, since most magnetic nanoparticles have a strong resistance to external stimuli, the effect of reducing the efficiency of destruction of materials in general magnetic hyperthermia can be considered insignificant. However, due to the unique magnetic properties of the magnetic nanoparticles, they are magnetized by an external magnetic field. As a result, there are some of retentive magnetism are remained in the particle and this retentive magnetism caused aggregation phenomenon. Moreover, several studies have reported that particle agglomeration has a negative effect on effective hyperthermia. Therefore, high dispersion stability is essential for efficient heat generation [42, 45]. Briefly, the cycle stability data show the mechanical strength of the Fe@Fe_3_O_4_ nanoparticles, and it is also evidence that the synthesized nanoparticles do not aggregate. In addition, the time required for the nanoparticles used in the previous studies to reach the maximum temperature of the application was about 10 min. However, in the case of our synthesized nanoparticles, the maximum temperature reached within 3 min. This result reveals that the patient can be treated with a short time of irradiation for magnetic hyperthermia.

## Conclusion

In this work, uniform Fe@Fe_3_O_4_ core–shell nanoparticles were synthesized by pyrolysis of iron pentacarbonyl followed by further controlled oxidation. The particles were coated with DMSA for water dispersion. The Fe@Fe_3_O_4_ nanoparticles show photothermal effect upper 20 °C after 5 min 1 W NIR irradiation and rate of temperature rise is maintained during 5 cycles. It is a similar or improved photothermal effect compared to other photothermal nanoparticles (Additional file 1: Table S3) [46]. Magnetic field-mediated heating property of the Fe@Fe_3_O_4_ nanoparticles is lower than other magnetic hyperthermia nanoparticles because of DMSA coating and relatively low frequency of magnetic field. In addition to these heating effects, the nanoparticles reported here show the advantages of low-cost, easy to synthesize. Therefore, the synthesized Fe@Fe_3_O_4_ nanoparticles are expected to be a good candidate for hyperthermia applications.

## Supplementary information

**Additional file 1: Fig. S1.** DLS measurements data of the DMSA coated Fe@Fe_3_O_4_ nanoparticles. Hydrodynamic disameter ( above ) were meassured as 18.45nm and its zeta potential ( below ) value is − 23.5mV due to thier carboxylate end. **Table S1.** Total heat generation and heat conversion efficiency calcuated by simple mathematical method and the result. **Table S2.** Table about the maximum temperature raise, total heat generation and efficiency from photothermal repeating test. **Figure S2.** (a) Fe@Fe_3_O_4_ in PBS after 48 h (left), redispersed Fe@Fe_3_O_4_ by simple agitation (right). (b) Photothermal characterization of the as-prepared Fe@Fe_3_O_4_ and Fe@Fe_3_O_4_ stored in PBS for 48 hours. The concentration is 10 mg/ml. **Table S3.** Comparison table between the as-prepared Fe@Fe_3_O_4_ nanoparticles and other photothermal nanoparticles reported in literatures.

## Data Availability

The datasets used and/or analyzed during the current study are available from the corresponding author on reasonable request.

## Abbreviations

DMSA: Dimercaptosuccinic acid
HAD: Hexadecylamine
DMSO: Dimethyl sulfoxide
PBS: Phosphate-buffered saline
TEM: Transmission electron microscopy
HR-TEM: High-resolution transmission electron microscopy
XRD: X-ray diffraction
XPS: X-ray photoelectron spectroscopy
VSM: Vibrating sample magnetometer
DLS: Dynamic light scattering
