# Supplementary material for: Magnetic and near-infrared derived heating characteristics of dimercaptosuccinic acid coated uniform Fe@Fe3O4 core–shell nanoparticles
Source: Nano Converg. 2020 Jun 8;7:20. doi: 10.1186/s40580-020-00229-4 (PMC7280462; doi:10.1186/s40580-020-00229-4)
Supplement: Supplementary file 1 — Additional file 1: Fig. S1. DLS measurements data of the DMSA coated Fe@Fe3O4 nanoparticles. Hydrodynamic disameter ( above ) were meassured as 18.45nm and its zeta potential ( below ) value is − 23.5mV due to thier carboxylate end. Table S1. Total heat generation and heat conversion efficiency calcuated by simple mathematical method and the result. Table S2. Table about the maximum temperature raise, total heat generation and efficiency from photothermal repeating test. Figure S2. (a) Fe@Fe3O4 in PBS after 48 h (left), redispersed Fe@Fe3O4 by simple agitation (right). (b) Photothermal characterization of the as-prepared Fe@Fe3O4 and Fe@Fe3O4 stored in PBS for 48 hours. The concentration is 10 mg/ml. Table S3. Comparison table between the as-prepared Fe@Fe3O4 nanoparticles and other photothermal nanoparticles reported in literatures. [file 40580_2020_229_MOESM1_ESM.docx]

**Supporting information:**

Magnetic and near-infrared derived heating characteristics of dimercaptosuccinic acid coated uniform Fe@Fe_3_O_4_ core-shell nanoparticles

*Changhyuk Koo^1†^, Hwichan Hong^1†^, Pyung Won Im ^2,3^, Hoonsub Kim^1^, Chaedong Lee^1^, Xuanzhen Jin^1^, Bingyi Yan ^1^, Wooseung Lee^1^ , Hyung-Jun Im^1^, Sun Ha Paek^2,3*^, Yuanzhe Piao^1,4*^*

*Correspondence: parkat9@snu.ac.kr (Y. Piao), paeksh@snu.ac.kr (S. H. Paek).
†Changhyuk Koo and Hwichan Hong contributed equally to this work.

^1^ Program in Nano Science and Technology, Graduate School of Convergence Science and Technology, Seoul National University, 145 Gwanggyo-ro, Yeongtong-gu, Suwon-si, Gyeonggi-do, 16229, South Korea
^2^ Department of Neurosurgery, Clinical Research Institute, Seoul National University Hospital, South Korea

^3^ Cancer Research Institute and Ischemia/Hypoxia Disease Institute, Seoul National University College of Medicine, Seoul, South Korea
^4^ Advanced Institutes of Convergence Technology, 145 Gwanggyo-ro, Yeongtong-gu, Suwon-si, Gyeonggi-do, 16229, South Korea


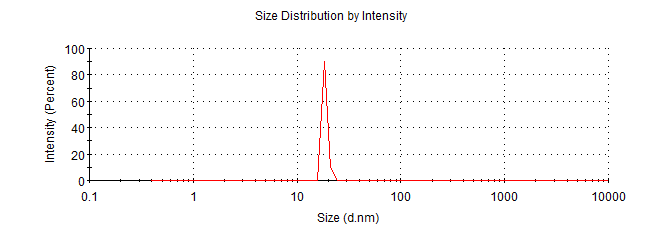


**Size : 18.45nm**


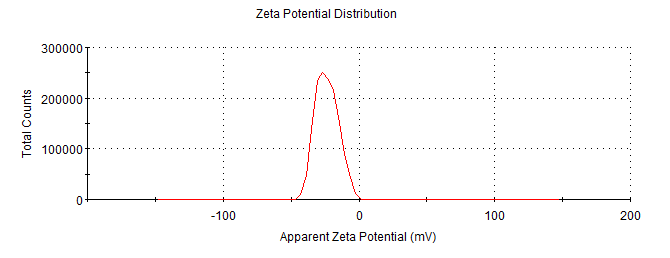


**Zeta : -23.50 mV**

**Fig. S1.** DLS measurements data of the DMSA coated Fe@Fe_3_O_4_ nanoparticles. Hydrodynamic disameter ( above ) were meassured as 18.45nm and its zeta potential ( below ) value is -23.5mV due to thier carboxylate end.

Heat energy generation = $\frac{C\Delta T}{dt}$
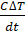


Conversion efficiency =$\frac{Heat energy generation}{Irradiated E (Watt)}$
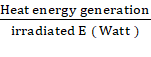


**Table S1.** Total heat generation and heat conversion efficiency calcuated by simple mathematical method and the result

Efficiency according to concentration

| C(mg/ml) | 10 | 2.5 | 0.625 | 0.156 |
| --- | --- | --- | --- | --- |
| Efficiency | 33.21% | 30.84% | 26.51% | 20.79% |
| Efficiency according to laser power | | | | |
| Laser P (W) | 1 | 1.5 | 2 |  |
| Efficiency | 33.21% | 30.70% | 28.40% |  |

**Table S-2.** Table about the maximum temperature raise, total heat generation and efficiency from photothermal repeating test.

|  | 1 | 2 | 3 | 4 | 5 | average  (expect 1) |
| --- | --- | --- | --- | --- | --- | --- |
| Maximum  delta T | 24.50 | 22.90 | 22.10 | 22.20 | 22.40 | 22.40 |
| Heat  generation(W) | 102.56 | 95.86 | 92.51 | 92.93 | 93.77 | 93.77 |
| Efficiency | 34.19% | 31.95% | 30.84% | 30.98% | 31.26% | 31.26% |


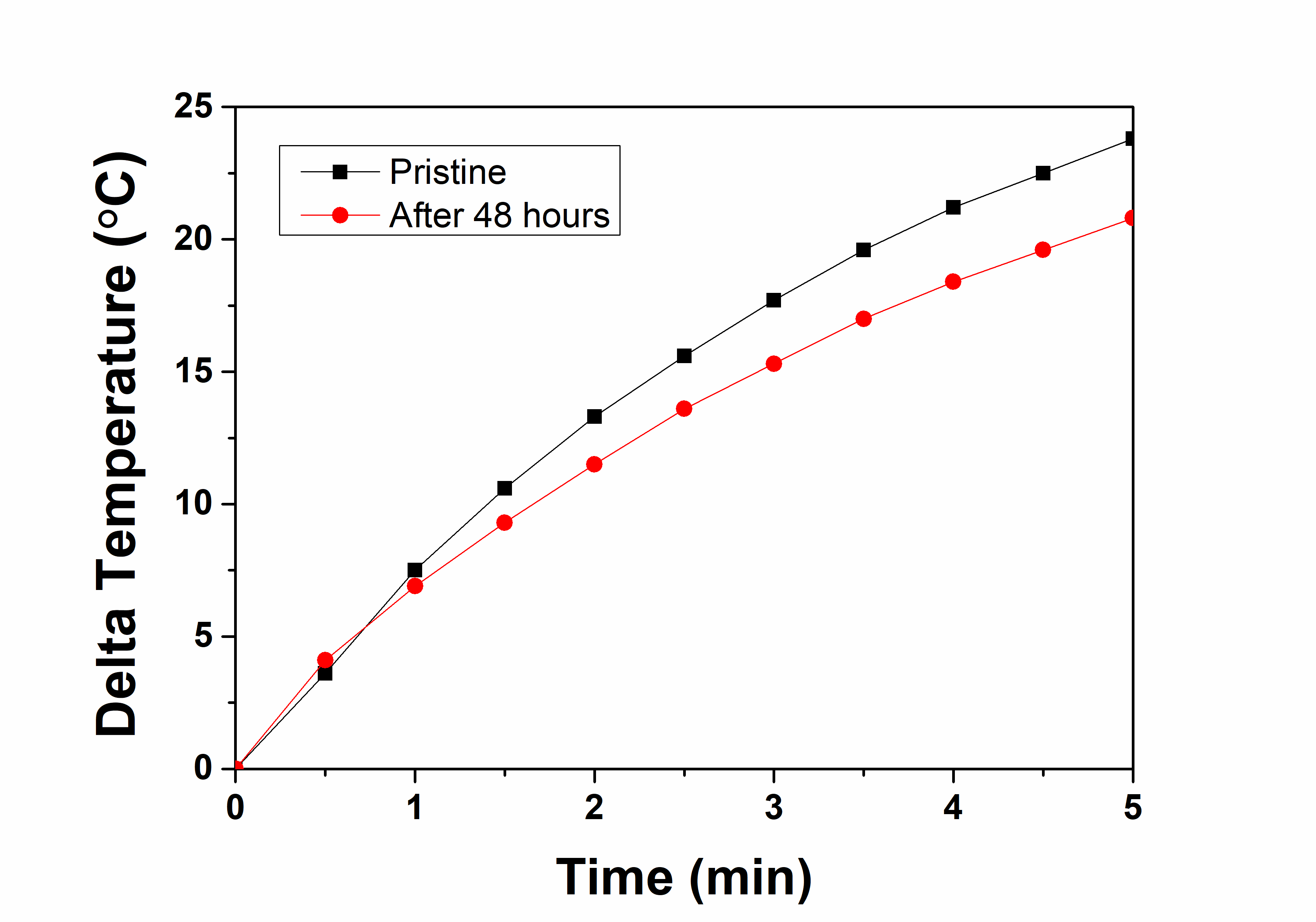

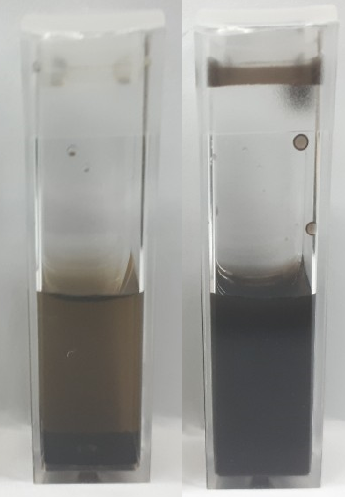


**(a)**

**(b)**

**Figure S2.** (a) Fe@Fe_3_O_4_ in PBS after 48hr (left), redispersed Fe@Fe_3_O_4_ by simple agitation (right). (b) Photothermal characterization of the as-prepared Fe@Fe_3_O_4_ and Fe@Fe_3_O_4_ stored in PBS for 48 hours. The concentration is 10 mg/ml.

**Table S3.** Comparison table between the as-prepared Fe@Fe_3_O_4_ nanoparticles and other photothermal nanoparticles reported in literatures.

| Nanoparticles | Size  (nm) | Concentration (mg/ml) | Irradiation power  (W/cm^2^) | Time (s) | Laser wavelength (nm) | Heating ΔT (℃) | ref |
| --- | --- | --- | --- | --- | --- | --- | --- |
| Fe_3_O_4_ | 10 | 0.08 | 0.25 | 600 | 808 | 13 | 5 |
| Fe_3_O_4_ nanocubes | 20 | 0.7 | 0.3 | 300 | 808 | 7 | 38 |
| Fe_3_O_4_-polydopamine | 20 | 0.1 | 1 | 600 | 808 | 24 | 39 |
| Gold nanorod | 40x10 | 0.8 | 2 | 600 | 808 | 38.8 | 46 |
| Fe@Fe_3_O_4_ | 15 | 0.625 | 1 | 300 | 808 | 19 | Present  work |
